# Viral suppression in adolescents on antiretroviral treatment: review of the literature and critical appraisal of methodological challenges

**DOI:** 10.1111/tmi.12656

**Published:** 2016-01-10

**Authors:** Rashida A. Ferrand, Datonye Briggs, Jane Ferguson, Martina Penazzato, Alice Armstrong, Peter MacPherson, David A. Ross, Katharina Kranzer

**Affiliations:** ^1^ London School of Hygiene and Tropical MedicineLondonUK; ^2^ World Health OrganizationGenevaSwitzerland; ^3^ Department of Public Health and PolicyUniversity of LiverpoolLiverpoolUK; ^4^ Department of Clinical SciencesLiverpool School of Tropical MedicineLiverpoolUK

**Keywords:** Adolescents, HIV, virological suppression, adolescents, VIH, suppression virologique

## Abstract

**Objective:**

Medication adherence is often suboptimal for adolescents with HIV, and establishing correct weight‐based antiretroviral therapy dosing is difficult, contributing to virological failure. This review aimed to determine the proportion of adolescents achieving virological suppression after initiating ART.

**Methods:**

MEDLINE, EMBASE and Web of Science databases were searched. Studies published between January 2004 and September 2014 including ≥50 adolescents taking ART and reporting on the proportion of virological suppressed participants were included.

**Results:**

From a total of 5316 potentially relevant citations, 20 studies were included. Only eight studies reported the proportion of adolescents that were virologically suppressed at a specified time point. The proportion of adolescents with virological suppression at 12 months ranged from 27 to 89%.

**Conclusion:**

Adolescent achievement of HIV virological suppression was highly variable. Improved reporting of virological outcomes from a wider range of settings is required to support efforts to improve HIV care and treatment for adolescents.

## Abstract (fr)

**Objectif:**

La compliance est souvent sous‐optimale chez les adolescents vivant avec le VIH et l’établissement correct du dosage de la thérapie antirétrovirale en fonction du poids est difficile, contribuant ainsi à l’échec virologique. Cette revue visait à déterminer la proportion d'adolescents atteignant une suppression virologique après initiation de l’ART.

**Méthodes:**

Recherches effectuées dans les bases de données MEDLINE, EMBASE et Web of Science. Les études publiées entre janvier 2004 et septembre 2014, comprenant ≥ 50 adolescents prenant l’ART et rapportant sur la proportion de participants avec une suppression virologique, ont été incluses.

**Résultats:**

Sur un total de 5316 citations potentiellement pertinentes, 20 études ont été incluses. Seules 8 études rapportaient sur la proportion d'adolescents avec une suppression virologique à moment donné. La proportion d'adolescents avec suppression virologique à 12 mois variait de 27% à 89%.

**Conclusion:**

L'atteinte de la suppression virologique du VIH chez l'adolescent était très variable. L'amélioration de la dissémination des résultats virologiques d'un éventail plus large d'endroits est nécessaire pour soutenir les efforts visant à améliorer les soins et le traitement du VIH pour les adolescents.

## Abstract (es)

**Objetivo:**

La adherencia a la medicación es a menudo subóptima para adolescentes con VIH y establecer una dosificación correcta de antirretrovirales basada en el peso es difícil, lo cual contribuye al fallo virológico. Esta revisión tenía por objetivo determinar la proporción de adolescentes que conseguían supresión virológica después de iniciar TAR.

**Métodos:**

Se realizaron búsquedas en las bases de datos MEDLINE, EMBASE y Web of Science. Se incluyeron los estudios publicados entre Enero 2004 y Septiembre 2014 incluyendo ≥ 50 adolescentes recibiendo TAR y que reportaban la proporción de participantes con supresión virológica.

**Resultados:**

De un total de 5316 citaciones potencialmente relevantes, se incluyeron 20 estudios. Solo 8 estudios reportaban la proporción de adolescentes que tenían supresión virológica en un punto de tiempo específico. La proporción de adolescentes con supresión virológica a los 12 meses variaba entre 27% ‐ 89%.

**Conclusión:**

Los logros de los adolescentes en cuanto a la supresión virológica del VIH eran altamente variables. Se requiere una mejora en la comunicación de los resultados virológicos de un amplio rango de lugares, que apoyen los esfuerzos para mejorar los cuidados y el tratamiento del VIH entre adolescentes.

## Introduction

An estimated 2.1 million adolescents (10–19 years of age) globally were living with HIV in 2012 1. HIV‐related deaths among adolescents are estimated to have tripled since 2000, making HIV the second leading cause of death among adolescents worldwide 2. Coverage of antiretroviral therapy (ART) is significantly lower (34%) in adolescents and children than in adults 3. With successful scale‐up of screening and treatment of infants infected with HIV, many of the >500 000 HIV‐infected children who started on ART during infancy are surviving to adolescence, often with complex treatment needs.

Adolescence is accompanied by rapid physical, psychological and physiological changes which influence health‐related behaviour. Adolescents frequently find consistent, long‐term medication adherence difficult, and HIV treatment is no exception 4, 5, 6. Maintaining sustained high levels of adherence to ART is the crux of successful treatment, preventing the development of drug resistance and disease progression, and decreasing risk of onward transmission once sexual debut occurs. Virological failure may also occur as a result of suboptimal drug dosing. Accurate weight‐based dosing is difficult to achieve during the growth spurt which occurs in adolescence, and frequent dose changes are necessary, which may be challenging in under‐staffed healthcare facilities in low‐resource settings, where the majority of HIV‐infected adolescents live, and can be confusing for the patient.

This review aimed to assess the proportion of adolescents taking ART in routine healthcare settings who achieve virological suppression in order to inform the need for interventions to optimise HIV outcomes in an age group that may be at high risk of treatment failure.

## Methods

This review was conducted in accordance with the standards of the Preferred Reporting Items for Systematic Reviews and Meta‐Analyses (PRISMA) group 7. A detailed protocol was prepared. We included cohort studies, randomised controlled trials (RCTs) and cross‐sectional studies of adolescents taking ART that investigated the prevalence and/or rate of virological suppression. Studies were included if they reported on least 50 HIV‐infected adolescents (aged 10–19 years) receiving ART, or where the mean or median age of participants was between 10 and 19 years. Studies where neither the mean nor the median age was reported, or where data were not disaggregated by age, were excluded. No exclusions with regard to language or geographical area were applied.

A compound search strategy was developed (Table S1), and the following electronic databases were searched: Medline (OVID), Embase (OVID), Global Health (OVID) and Web of Science. Reference lists of all studies identified by the above methods, and bibliographies of relevant systematic reviews or meta‐analyses were examined. All references were imported into EndNote (Thompson Reuters), and duplicates were removed. Titles and abstracts were examined independently by two reviewers (RF and KK) to exclude studies that clearly did not meet inclusion criteria.

The full text of all potentially relevant studies was obtained, and the inclusion criteria were applied using a pre‐tested eligibility form. Data extraction was independently performed by two reviewers (RF and KK) using a standardised data extraction form. The following information was obtained for included studies: start and end dates of the study; study design; location (country, healthcare setting); rate/proportion of participants who died or were lost to follow‐up; age; sex; CD4 cell count at ART initiation; ART regimen; median duration of follow‐up; median duration on ART; laboratory method for HIV viral load measurement; viral load threshold used to define suppression; proportion of participants with viral load ascertained; proportion of participants with virological suppression at 12, 24, 36 months; and overall proportion with virological suppression.

Primary outcomes were proportions of adolescents with suppressed viral load at 12 and 24 months of starting ART. Secondary outcomes included proportion with virological suppression irrespective of time on ART and rate of virological suppression. Virological suppression was defined as by the definitions used by the authors of the study.

A modified Newcastle‐Ottawa Scale was used to assess the risk of bias 8. A scoring scale to evaluate potential sources of bias was used. It included risk of selection bias; method of viral load testing; recording of baseline characteristics; duration of ART and follow‐up; proportion of participants with missing outcomes; and appropriateness of analysis. Studies were scored on each these criteria with studies graded as providing good (score 7–8), moderate (score 5–6) or low quality of evidence (3–4), or serious risk of bias (<3).

## Results

From a total of 5316 potentially relevant unique citations, 20 studies were included in this review (Figure S1). Of the 20 studies that were included, 7 (35%) were from Africa 9, 10, 11, 12, 13, 14, 15, 8 (40%) were from North America 16, 17, 18, 19 and Europe 20, 21, 22, 23, 3 (15%) were from Asia 24, 25, 26 and 2 (10%) were from Latin America 27, 28 (Table 1). Half (*n *= 10) were cohort studies, and half were cross‐sectional studies (*n *= 10). The studies were predominantly based on multicentre and/or multicountry research cohorts, or from urban, specialist centres. The cohorts included perinatally, sexually and parenterally infected adolescents.

**Table 1:** Baseline characteristics of studies included in the review

Author, year	Study period	Study setting	Study design	N	Age range (median age)	% Female	Mode of HIV acquisition	Median CD4 count at initiation (IQR), cells/μl	Median HIV viral load (log_10_) at initiation (IQR)
Africa									
Bakeera‐Kitaka (2008) 9	2004–2006	Uganda; Paediatric HIV Clinic, Mulago Hospital Public sector	Cohort	118	10–19 years (13.6) at ART initiation	64%	Majority perinatal	124 (12–249)	5.4 (4.9–6.8)
Evans (2013) 10	2004–2010	South Africa; Public sector HIV clinics; 5 urban Gauteng province; 2 rural Mpumalanga	Cohort	652	10–19 years at ART initiation	67%	Mixed	109 (24–195):10–14 years 133 (54–198): 15–19 years	NR
Nglazi (2012) 11, a	2002–2009	Cape Town, South Africa; Public sector community‐based ART programme^a^^,^ b	Cohort	65	9–19 years (11.5) at cohort entry in 2009	66%	Mixed: 72% perinatal, 6% sexual, 2% other	134 (41–198)	4.8 (4.5–5.2)
Nachega (2009) 12	1999–2006	9 countries in southern Africa; Private‐sector, employer‐subsidised disease management programme ‘Aid for AIDS’	Cohort	154	11–19 years (16.4) at ART initiation	73%	Sexual	144 (27–246)	5.1 (4.5–5.6)
Mutwa (2014) 13	2009–2010	Kigali, Rwanda; University Teaching hospital	CS	424	1.7–18.8 years (10.8) at time of study	52%	Perinatal	NR	NR
Sebunya (2013) 14	2004–2009	Kampala, Uganda: JCRC‐HIV care and research institution	Cohort	236	10–18 years at ART initiation	NR	Majority perinatal	135 (50–210): Unsuppressed 130 (44–262): Suppressed	NR
Cutsem (2010) 15	2000–2007	Cape Town, South Africa; Public sector primary care clinics supported by MSF	Cohort	86	10–19 years at ART initiation	NR	NR	127 (68–183)	NR
Asia									
Chokephaibulkit (2014) 25	2011	18 clinics in 6 Asian countries: TApHOD; public or university‐based paediatric HIV referral clinics; urban/semi‐urban	CS	987	12–18 years at the last clinic visit		Perinatal	160 (30–337): 12–14 years 98 (22–255) >>15 years	NR
Shet (2013) 26	NR	Bangalore, India; tertiary care paediatric clinic	CS	80	2–16 years (10) at time of study	38%	Perinatal	275	NR
Zhao 24 (2011) b	2006–2011	Henan province, China; rural national programme	CS	245	11–16 years (13.9) at time of study	33%	79% perinatal, 20% parenteral	NR	NR
South and Central America									
Santos Cruz (2011) 28	2002–2006	15 sites in Brazil, Mexico and Argentina; NISDI cohort	Cohort	120	12–21 years (at time of enrolment into cohort)	55%	58% perinatal, 28% sexual, 8% transfusion, 6% unknown	NR	NR
Santos Cruz (2014) 27	2009–2011	5 regions in Brazil	CS	57	13–18 years at time of study	NR	Perinatal	NR	NR
North America									
Murphy (2005) 16		13 USA cities; REACH cohort	CS	231	15–22 years (18.4) at time of study	73%	Sexual	NR	NR
Chandwani (2012) 17	2003–2005	5 clinics in Washington/Baltimore /New York; Adolescent Impact Study	CS	104	13–21 years (16.4) at time of study	54%	75% perinatal, 25% other	NR	NR
Van Dyke (2011) 18	2007–2009	15 sites USA; PHACS AMP Cohort	CS	451	7–16 years (12.2) at time of study	53%	Perinatal	CD4% 19 (12.25)	NR
Flynn (2007) 19	1999–2001	USA: multicentre research cohort PACTG 381	Cohort	120	11–22 years at ART initiation	Approx. equal	Sexually‐infected	NR	NR
Europe									
De Mulder (2012) 20	1997–2011	Madrid Cohort, Spain. Study at time of transfer to adult services	CS	112	Mean age: 18.9 years at time of study	54%	94% perinatal	<200: 64 (57%) 200–499: 37 (33%) >500: 11 (10%)	NR
Cohere (2008) 21	1998–2006	30 European countries	Cohort	201	13–17 years (16.5) at time of study	63%	28% perinatal, 38% heterosexual, 4% IVDU, 3% gay	222 (110, 340)	4.8 (4.0, 5.2)
Dollfus (2010) 22		90 health centres, France; EPF/ANRS C010 cohort	CS	210	10–17 years (15) at time of study	50%	Perinatal	NR	NR
Judd (2007) 23	1997–2006	UK/Ireland Centres: CHIPS Cohort	Cohort	141	≥10 years at ART initiation	NR	NR	NR	NR

CD4 and VL at initiation of ART, except Nglazi: CD4/VL at cohort entry.

CS, Cross‐sectional; ART, antiretroviral therapy; IVDU, intravenous drug user; NR, Not reported, JCRC, Joint Clinical Research centre, MSF, Medicine Sans Frontieres, TApHOD, Treat Asia Pediatric HIV Observation Database, NISDI, NICHD International Site Development Initiative, REACH, Reaching for Excellence in Adolescence Care and Health, PHACS AMP, Pediatric HIV/AIDS Cohort Study. Adolescent Master Protocol, EPF/ANRS, French Perinatal Cohort, CHIPS, Collaborative HIV Pediatric Study.

a Adolescent‐centred service (Nglazi: introduced 2008).

b Age, mode of acquisition, gender, duration of ART refer to only the 76 participants of 245 who had virological failure.

Only eight studies reported the proportion of adolescents that were virologically suppressed at a specified time point: six 9, 10, 11, 12, 21, 23, two 12, 15 and one 19 study reported the proportion who were virologically suppressed at 12, 24 and 36 months after ART initiation, respectively (Table 2). The remainder of the studies provided an overall proportion of virologically suppressed adolescents among those in care, without taking the duration of ART treatment into account.

**Table 2:** Virological outcomes in HIV‐infected adolescents treated with antiretroviral therapy (ART)

Study	ART history & regimen	Median duration of follow‐up	Median duration of ART	Virological suppression cut‐off	Proportion of participant with viral load ascertained, %	Proportion virological suppressed, %			Overall proportion with virological suppression, %	Study quality
						12 months	24 months	36 months		
Bakeera‐Kitaka (2008) 9	Naive; 96% NNRTI‐based	NR	NR	<400	72	89				***
Evans (2013) 10, a	Naive; 99% NNRTI‐based	NR	23.9 months: 10–14 years 15.6 months: 15–19 years	<400	21	76				***
Nglazi (2012) 11, b	Naive; 99% NNRTI‐based	34.6 months	NR	<400	52	27				***
Nachega (2009) 12, c	Naive; 92% NNRTI‐based	27 months	NR	<400	45, 25	46	44			***
Mutwa (2014) 13	On treatment; 99% NNRTI‐based	3.4 years	NR	<40	85				61	***
Sebunya (2013) 14	On treatment; 100% NNRTI‐based	NR	NR	<400	67				63	***
Cutsem (2010) 15	Naive; NR	NR	NR	<400	NR		87			**
Chokephaibulkit (2014) 25	86% on first line treatment	NR	6.0 years (4.3–7.5)	<400	84				87	***
Zhao (2011) 24	On treatment; 100% NNRTI‐based	NR	33.1 months	<50	96				52	***
Shet (2013) 26	On treatment; 100% NNRTI‐based	NT	31 months	<400	82				85	***
Santos Cruz (2011) 28	Treatment experienced; NR	34 months: vertically infected 35 months: horizontally infected	NR	<400	97				37.5	**
Santos Cruz (2014) 27	Treatment experienced: NR	NR	7 years (mean)	<50	53				49	**
Flynn (2007) 19	Treatment experienced: NR	NR	NR	<400	37			66		*
Chandwani (2012) 17	Treatment experienced: NR	NR	21% on ART for < 1 years	<400	NR				28	*
Murphy (2005) 16	Treatment experienced: NR	NR	NR	<10	100				69	**
Van Dyke (2011) 18	Highly treatment experienced; NR	NR	NR		NR				68	**
De Mulder (2012) 20	NR: Highly treatment experienced	15.6 years	11.5 years	<200	100				47	***
Judd (2007) 23	Naive; NR	NR	NR	<400	53	86				*
Dollfus (2010) 22	Highly treatment experienced; NR	NR	NR	<50	96				43	**
Cohere (2008) 21	Highly treatment experienced; NR	2.2 years		<50	NR	46				**

‘Naive’, not on treatment at start of study; NR, not reported.

Study quality: ****: good, ***: moderate, **: low, *: serious risk of bias.

a 33.7% changed regimens: 5% virological failure; 11% toxicity; 6% non‐adherence; 6% pregnancy).

b Treatment failure rate (VL suppression followed by 2 subsequent VL >1000 copies/ml): 8.2/100py.

c Viral rebound after suppression 42% at 12 months (only among those with initially suppressed VL).

In the six studies that reported this, the proportion of adolescents with virological suppression at 12 months after ART initiation varied from 27% to 89% (Table 2). Virological suppression rates in studies not stratified by duration on ART ranged between 28% and 87%. Approximately one‐third of studies (*n *= 8) neither reported on the median duration on ART, nor on the median duration of follow‐up 9, 14, 15, 16, 18, 19, 22, 23. Four studies 15, 17, 18, 21 did not record the completeness of outcome data, and two studies had a very high proportion of missing viral load data (79% and 63%) 10, 19. Overall most studies were scored as being moderate or low quality (Table 2).

## Discussion

The main finding of this study was the paucity of data on virological outcomes among adolescents with HIV, despite more than 15 years of availability of combination ART. In included studies, there was substantial variation in the proportion of adolescents achieving virological suppression, and accurate overall estimates of the effectiveness of ART on viral suppression in this age group cannot be drawn because of this variability and the low to moderate quality of most studies. Thus, a meta‐analysis providing a pooled effect estimate was not attempted.

The studies included in this review illustrate some of the specific methodological issues encountered when analysing ART outcomes in children and adolescents, but also more general issues around analysis and reporting of ART outcomes. Any observational cohort study should be reported according to the STROBE (strengthening the reporting of observational studies in epidemiology) checklist, which has been endorsed by many high impact journals 29, 30. The STROBE checklist recommends that for each explanatory and outcome variable, the source of data and the methods of assessment (measurement) are described, the number of individuals at each stage of the study is clearly reported and the time of follow‐up is provided. Furthermore, the number of participants with missing data should be recorded and confounders taken into account 31. Most of the studies included in this review did not provide all this information, hindering our ability to accurately identify the proportion of adolescents succeeding on ART. The responsibility of accurate reporting lies not only with the authors, but also with the wider scientific community including journal editors and peer reviewers. Some of the studies included in this review were published before the STROBE checklist was developed.

Even more critical is the fact that a number of the included cross‐sectional studies provided point estimates of viral load suppression without stratifying the results by time on ART. Several of these studies did not even report the mean or median time on ART 14, 16, 17, 18, 22. Time on ART is highly likely to act as a confounder and cohorts with similar success rates, but different follow‐up times will appear to have different virological suppression rates. Thus, unstratified point prevalence estimates of virological suppression are meaningless, and we urge researchers to report results stratified by duration on ART in all future studies.

All included studies classified participants as adolescents according to their age at initiation of ART. A child starting ART on their eighth birthday will enter adolescence two years after ART initiation, whereas a child who starts ART at 6 months of age will have been on ART for 9.5 years before entering adolescence. Age‐updated analysis is therefore especially important when examining outcomes in children and adolescents. None of the studies included in the review attempted an age‐updated analysis. In practice, clinicians want to know the likelihood of virological suppression of a 14‐year old who has been taking ART for a few months, several years or over a decade. Clinicians are unlikely to assess patients aged 14 on the basis of having started ART as a child aged 1 year, 8 years or as adolescents aged 13. This information can only be obtained if age‐updated analyses are performed, alongside the analyses stratified by time on treatment discussed above.

A total of 21 studies included more than 50 adolescents on ART and reported on virological suppression, but did not provide adolescent‐specific suppression estimates but analysed adolescents together with children, which meant they had to be excluded. These studies could have contributed substantially to the body of evidence if age‐specific estimates had been provided. Furthermore, systematic reviews focus on peer‐reviewed published studies and thus are subject to publication bias. Due to heterogeneity of study designs and outcome definitions, summary estimates could not be calculated. Viral load testing methods have evolved over time, which makes comparison of studies across different periods and settings challenging. Further limitations of this review are the quality of studies included and high levels of missing data in some studies.

In conclusion, most available estimates of viral load suppression in adolescents are not very informative due to serious methodological concerns. Authors, journals and peer reviewers should be encouraged to follow the recommendation for observational cohort studies as laid out by the STROBE statement. We recommend the development of guidelines for ART cohort reporting focused on children, and adolescents to ensure data are analysed using generally accepted age groups such as 0, 1–4, 5–9, 10–14, 15–19 years, that time on ART is taken into account through stratified analyses, and that age‐updated analyses are conducted.

## Supporting information


**Figure S1** Selection process for the inclusion of studies.
**Table S1** Search strategy for Medline, Embase and Global Health.
