# Supplementary material for: Viral suppression in adolescents on antiretroviral treatment: review of the literature and critical appraisal of methodological challenges
Source: Trop Med Int Health. 2016 Jan 10;21(3):325–33. doi: 10.1111/tmi.12656 (PMC4776345; doi:10.1111/tmi.12656)
Supplement: Supplementary file 1 — Figure S1 Selection process for the inclusion of studies. Table S1 Search strategy for Medline, Embase and Global Health. [file TMI-21-325-s001.docx]

**Supplement**

**
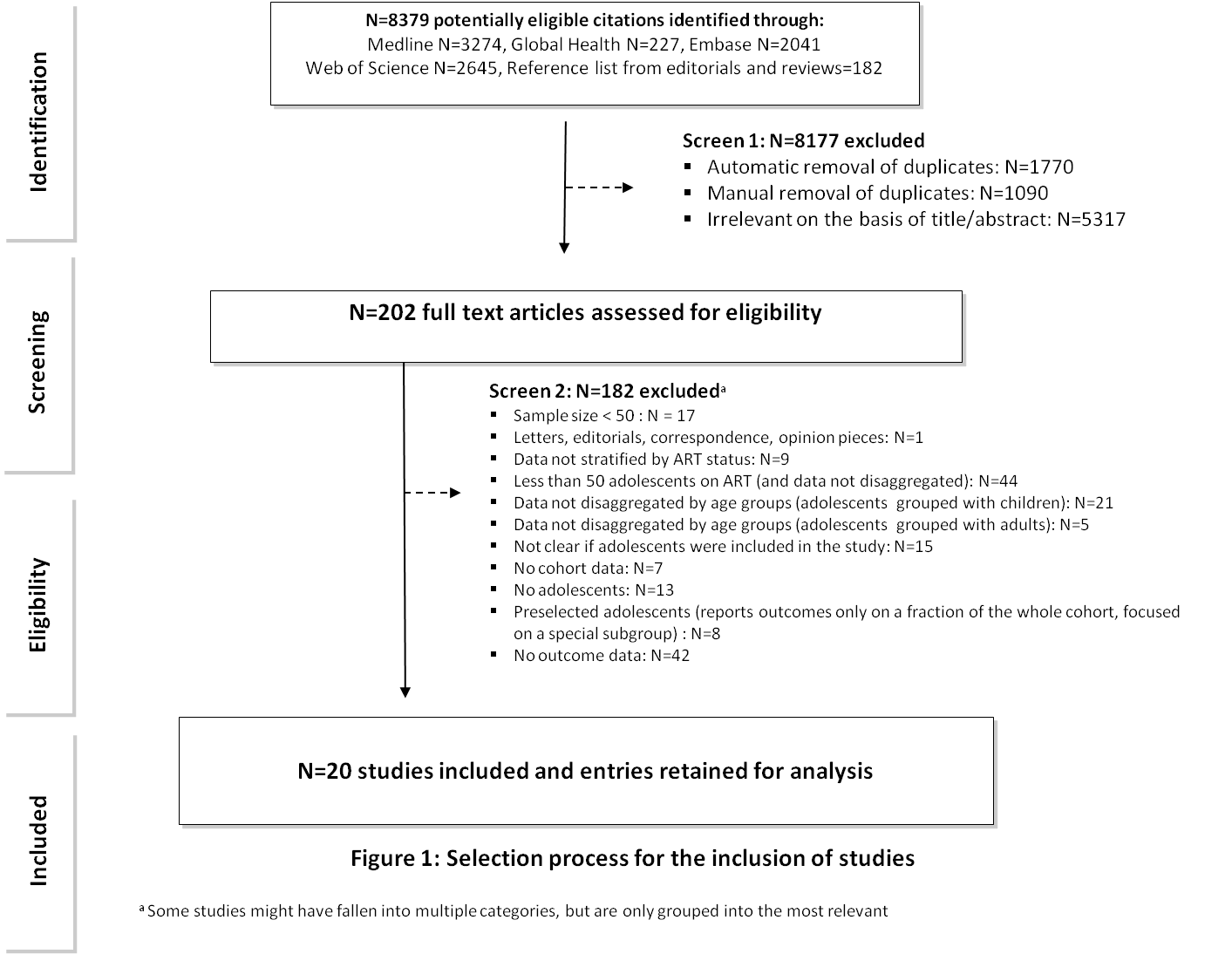
**

**Supplement**

**Search strategy for Medline, Embase and Global Health**

| **SET** | **TOPIC** | **SEARCH TERMS** |
| --- | --- | --- |
| 1 | Adolescents | ADOLESCENT |
| 2 |  | CHILD |
| 3 |  | YOUNG ADULT |
| 4 |  | HOMELESS YOUTH |
| 5 |  | Adolescent* |
| 6 |  | Young adj1 adult* |
| 7 |  | Minor* |
| 8 |  | Young adj1 person |
| 9 |  | Young adj1 people |
| 10 | Set 1-9 were combined with "or" | |
| 11 | HIV | HIV |
| 12 |  | HIV ANTIBODIES |
| 13 |  | HIV-2 |
| 14 |  | HIV-1 |
| 15 |  | HIV SEROPOSITIVITY |
| 16 |  | HIV LONG-TERM SURVIVORS |
| 17 |  | ACQUIRED IMMUNODEFICIENCY SYNDROME |
| 18 |  | hiv |
| 19 |  | aids |
| 20 | Set 11-19 were combined with "or" | |
| 21 | ART | ANTI-RETROVIRAL AGENTS |
| 22 |  | ANTI-HIV AGENTS |
| 23 |  | ANTIRETROVIRAL THERAPY, HIGHLY ACTIVE |
| 24 |  | art |
| 25 |  | arv |
| 26 |  | haart |
| 27 |  | antiretroviral adj1 therapy |
| 28 |  | antiretroviral adj1 treatment |
| 29 | Set 21-28 were combine with or | |
| 30 | Cohort | COHORT STUDIES |
| 31 |  | LONGITUDINAL STUDIES |
| 32 |  | cohort* |
| 33 |  | longitudinal |
| 34 | Set 30-33 were combined with “or” | |
| 35 | Set 10, 20, 29, 34 were combined with “or” | |

Words written in capital letters were used as MeSH headings, the others were used as free text. Search terms which were the same for all databases are only mentioned once under the EMBASE heading

**Search strategy for Web of Science**

Set 1 (adolescent*or youth*) or (young adult*) or (young adult*)

Set 2 hiv or aids

Set 3 (antiretroviral*) or haart or (arv*) or (art*)

Set 4 (cohort*) or (longitudinal) or (prospective follow-up)

Set 1-4 combined with “or”
